# Prevalence and impact of sarcopenia in individuals with heart failure with reduced ejection fraction (the SARC-HF study): A prospective observational study protocol

**DOI:** 10.1371/journal.pone.0300918

**Published:** 2024-03-21

**Authors:** Pablo Marino Corrêa Nascimento, Luiz Fernando Rodrigues Junior, Mauro Felippe Felix Mediano, Valéria Gonçalves da Silva, Bernardo Rangel Tura, Fabio César Sousa Nogueira, Gilberto Domont, Adriana Bastos Carvalho, Antônio Carlos Campos de Carvalho, Taís Hanae Kasai-Brunswick, Claudio Tinoco Mesquita, Humberto Villacorta Junior, Helena Cramer Veiga Rey

**Affiliations:** 1 National Institute of Cardiology, Rio de Janeiro, Brazil; 2 Fluminense Federal University, Niterói, Brazil; 3 Federal University of the State of Rio de Janeiro, Rio de Janeiro, Brazil; 4 Oswaldo Cruz Foundation, Rio de Janeiro, Brazil; 5 Federal University of Rio de Janeiro, Rio de Janeiro, Brazil; University of Pisa, ITALY

## Abstract

Sarcopenia, a clinical syndrome primarily associated with reduced muscle mass in the elderly, has a negative impact on quality of life and survival. It can occur secondarily to other diseases such as heart failure (HF), a complex clinical syndrome with high morbidity and mortality. The simultaneous occurrence of these two conditions can worsen the prognosis of their carriers, especially in the most severe cases of HF, as in patients with reduced left ventricular ejection fraction (LVEF). However, due to the heterogeneous diagnostic criteria for sarcopenia, estimates of its prevalence present a wide variation, leading to new criteria having been recently proposed for its diagnosis, emphasizing muscle strength and function rather than skeletal muscle mass. The primary objective of this study is to evaluate the prevalence of sarcopenia and/or dynapenia in individuals with HF with reduced LVEF according to the most recent criteria, and compare the gene and protein expression of those patients with and without sarcopenia. The secondary objectives are to evaluate the association of sarcopenia and/or dynapenia with the risk of clinical events and death, quality of life, cardiorespiratory capacity, ventilatory efficiency, and respiratory muscle strength. The participants will answer questionnaires to evaluate sarcopenia and quality of life, and will undergo the following tests: handgrip strength, gait speed, dual-energy X-ray absorptiometry, respiratory muscle strength, cardiopulmonary exercise, as well as genomic and proteomic analysis, and dosage of N-terminal pro-B-type natriuretic peptide and growth differentiation factor-15. An association between sarcopenia and/or dynapenia with unfavorable clinical evolution is expected to be found, in addition to reduced quality of life, cardiorespiratory capacity, ventilatory efficiency, and respiratory muscle strength.

## Introduction

Heart failure (HF) is defined as a complex syndrome resulting from any structural or functional impairment of ventricular filling or blood ejection [1], which progresses with high mortality [2], is comparable to several types of neoplasms [3], and leads to a considerable reduction in quality of life [4].

The term “sarcopenia” was first employed by Irwin Rosenberg in 1988 [5,6], although sarcopenia was formally recognized as a muscle disease only in 2016, by receiving a code (M62.84) in the International Classification of Diseases, Tenth Revision, Clinical Modification (ICD-10-CM) [7]. Sarcopenia was originally defined as an “involuntary loss of skeletal muscle mass and consequently strength” [8]. However, diagnostic criteria have evolved over the past few decades, and the focus has shifted from an isolated reduction in skeletal muscle mass [9] to a reduction in muscle strength or function, whether associated [10,11] with reduced muscle mass. In fact, there is much more consistent evidence that dynapenia (a condition characterized by reduced muscle strength independently of loss of muscle mass, not caused by neurologic or muscular diseases) contributes more significantly to relevant outcomes such as the risk of physical disability, poor physical performance, and mortality, than an isolated reduction of muscle mass. This possibly happens because the reduction of muscle mass does not fully explain the reduction of muscle strength, which also depends on neuromotor properties [12].

Over the last few years, several criteria for the diagnosis of sarcopenia have been proposed by numerous international societies, the most recent being those of the European Working Group on Sarcopenia in Older People (EWGSOP2) [13] and the Sarcopenia Definition and Outcomes Consortium (SDOC) [11,14] (Table 1).

**Table 1:** Diagnostic criteria for sarcopenia.

Society	Year	Sarcopenia	Severe Sarcopenia
EWGSOP2 [13])	2019	ASMMi: < 7.0 kg/m^2^ (M); <5.5 kg/m^2^ (W); HGS: < 27.0 kg (M); < 16.0 kg (W) **or** FTSS > 15 s	ASMMi: < 7.0kg/m^2^ (M); <5.5 kg/m^2^ (W); HGS: < 27.0kg (M); < 16.0kg (W) **or** FTSS > 15 s; GS: ≤ 0.8 m/s
SDOC [11,14]	2020	HGS: < 35.5 kg (M); < 20.0 kg (W); GS: < 0.8m/s	—

ASMMi, appendicular skeletal muscle mass index; EWGSOP2, European Working Group on Sarcopenia in Older People; FTSS, five times sit-to-stand test; GS, gait speed; HGS, handgrip strength; M, men; SDOC, Sarcopenia Definition and Outcomes Consortium; W, women.

Sarcopenia is a relevant clinical condition because it is associated with increased risk of falls [15] and fractures [15], impaired ability to perform activities of daily living [16], cognitive deficit [17], reduced mobility [18], functional decline [19], and loss of independence [20], with consequent need for long-term care [21] and reduced quality of life [22]. Sarcopenia is also associated with depression [15], cardiometabolic syndrome [23], respiratory diseases [24], and increased mortality [19]. From the economic point of view, it increases the risk of hospitalization and the cost of treatment during hospital stays [25].

Sarcopenia is classified as primary when associated with aging, and secondary when identified in the context of chronic diseases, such as malignant neoplasms, chronic obstructive pulmonary disease, chronic renal disease, and HF [26].

Individuals with both HF and sarcopenia, when compared with those without sarcopenia, have reduced muscle strength [27], poorer quality of life [28], lower values of peak exercise oxygen consumption (peak VO_2_) [27], distance walked in the six-minute walk test [27], and gait speed (GS) in the four-meter walk test (4mWT) [27], in addition to higher mortality [29]. Moreover, the mere reduction in muscle strength alone, even when unaccompanied by a change in muscle mass, is associated with higher mortality in individuals with HF [30,31]. However, due to the heterogeneous diagnostic criteria for sarcopenia used in the literature before it was classified as a disease, estimates of its prevalence present a wide variation, especially when secondary to HF.

## Materials and methods

### Study objective

The primary objective of the present study is to evaluate the prevalence of sarcopenia and/or dynapenia in individuals with heart failure associated with reduced ejection fraction (HFrEF), according to the most recent criteria [11,13,14], and compare the gene and protein expression in those with and without sarcopenia and/or dynapenia, seeking new therapeutic targets for HFrEF.

The secondary objectives are to investigate the association of sarcopenia and/or dynapenia with the risk of clinical events such as hospitalization for HF, heart transplantation or ventricular assist device (VAD) implantation, and with factors including death, quality of life, cardiorespiratory capacity, ventilatory efficiency, and respiratory muscle strength.

### Hypothesis

The presence of dynapenia and/or sarcopenia is associated with reduced respiratory muscle strength, reduced aerobic power, and ventilatory inefficiency in individuals with HFrEF, having a negative impact on these individuals’ quality of life and prognosis. The analysis of molecular factors by genomics and proteomics can help identify clinical prediction markers and new therapeutic targets.

### Study design and setting

This is a multicenter longitudinal observational study including individuals with HFrEF referred at outpatient clinics at the National Institute of Cardiology (INC), the Pedro Ernesto University Hospital (HUPE) of Rio de Janeiro State University, and the Antônio Pedro University Hospital (HUAP) of Fluminense Federal University, all located in or around the city of Rio de Janeiro, Brazil.

The baseline assessments will be carried out in two visits, conducted at the premises of INC, HUPE, and HUAP and at the Biomedical Institute of the Federal University of the State of Rio de Janeiro (UNIRIO).

The longitudinal follow-up of participants will take place during periodic reevaluation consultations every six months during five years. Information on deaths, hospital admissions, cardiac transplantation, or VAD implantation, elective and urgent or emergency, will be collected during the follow up interviews and from medical records by the research team. Data regarding mortality outside the hospitals included in the study will be extracted from the Rio de Janeiro judicial court database (http://www4.tjrj.jus.br/Portal-Extrajudicial/CNO/). The time until the occurrence of each of the clinical outcomes will be determined, taking the participant’s entry into the study as the starting date.

This cohort study was designed following the recommendations of the Strengthening the Reporting of Observational Studies in Epidemiology (STROBE).

### Ethics

Participants will be informed of the nature of the study and, if agree to participate, they will be asked to sign an informed consent form. The Helsinki Declaration of Rights (October 2013 version) and Resolution 466/2012 of the Brazilian National Health Council will be respected. The study was approved by the INC Research Ethics Committee under Certificate of Presentation for Ethical Appraisal (CAAE) No. 50974021.3.1001.5272. The results of the exams performed will be sent to the physicians caring for the participants included in the study.

### Inclusion criteria

Individuals diagnosed with HF by the Framingham criteria [32], with left ventricular ejection fraction (LVEF; assessed by echocardiography using Simpson’s method) below 40%, of both sexes, aged over 18 years, under outpatient treatment, and clinically stable in the previous six months will be included.

### Exclusion criteria

Individuals with severe musculoskeletal or neurological alterations that prevent them from performing the handgrip strength (HGS) and GS tests, and pregnant women will be excluded. The cardiopulmonary exercise test (CPET) will not be performed in individuals with contraindications for this test, such as unstable angina with precordial pain at rest, decompensated heart failure, uncontrolled cardiac arrhythmias, decompensated metabolic disorders, febrile or acute systemic illness, severe stenotic valve lesions, obstructive hypertrophic cardiomyopathy, pregnancy, or any severe musculoskeletal alteration that prevents the participant from walking on the treadmill [33]. However, patients with contraindications for CPET will still be able to undergo all other procedures and will not be excluded from the study.

### Study procedures

**Fig 1:**
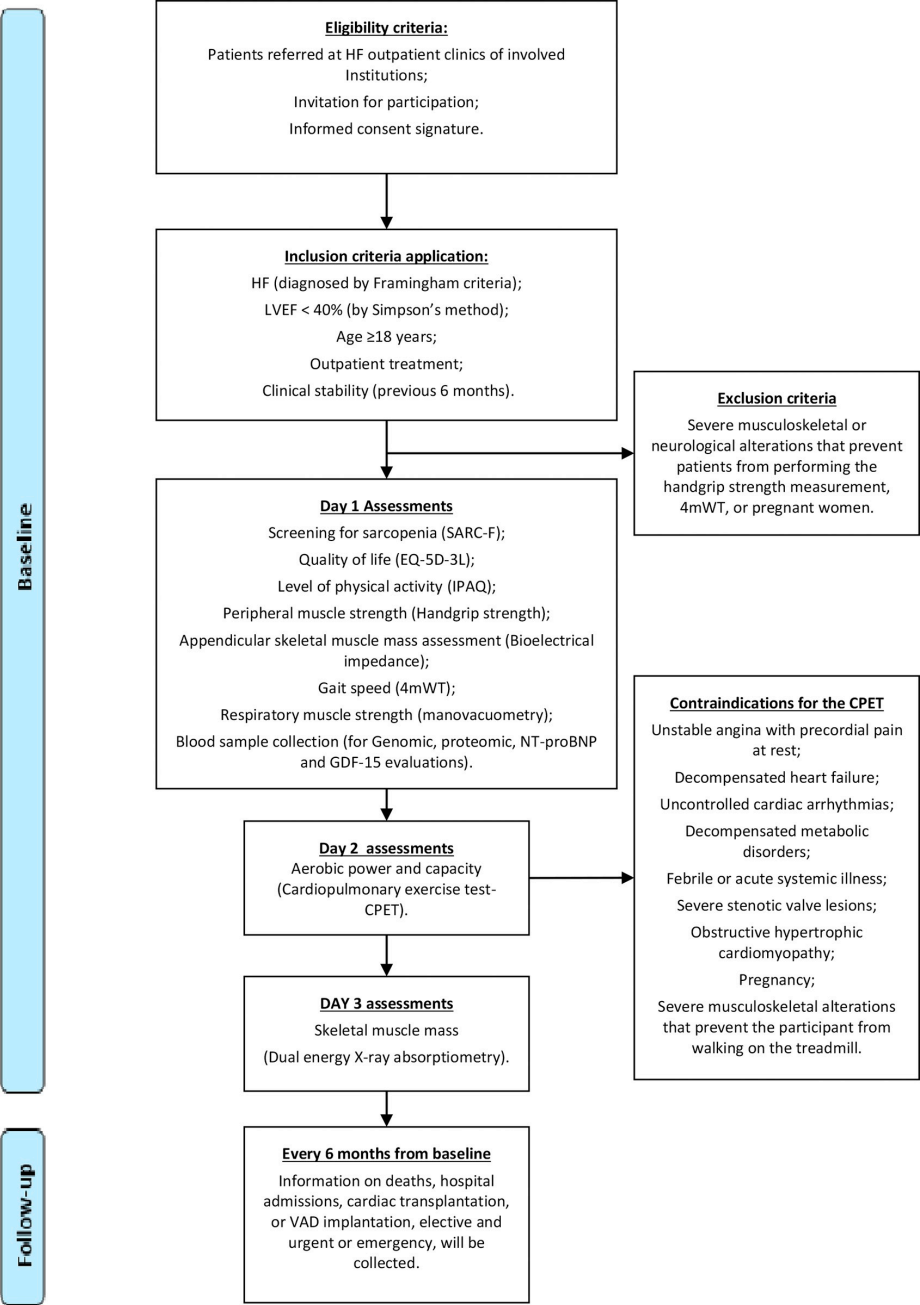
Flowchart of the study.

The examinations and procedures that will be performed in the study are schematically represented in Fig 1.

### Screening for sarcopenia

For the tracking of sarcopenia and subsequent comparison with the EWGSOP2 [13] and SDOC [11,14] diagnostic criteria, the SARC-F questionnaire [16] will be used. This questionnaire includes questions about five components: strength, assisted walking, getting up from a chair, climbing stairs, and falls in the previous year. The total SARC-F score ranges from 0 to 10—that is, from 0 to 2 points for each of the components, with 0 being the best score and 10 the worst. A score equal to or greater than 4 is associated with deficit of instrumental activities of daily living, lower HGS, GS less than 0.8 m/s, greater slowness to get up from the chair, and greater risk of hospitalization and death [16], with low to moderate sensitivity and very high specificity to predict reduced muscle strength [34]. In the present study, the version validated in Portuguese will be used [35].

### Quality of life

The quality of life will be evaluated by the EQ-5D-3L instrument [36]. This instrument is based on a health classification defined by a descriptive system with five dimensions (mobility, self-care, usual activities, pain or discomfort, and anxiety or depression), with three levels of answers for each dimension (“no problems,” “some problems,” and “extreme problems”).

### Level of physical activity

The level of physical activity will be determined by applying the International Physical Activity Questionnaire (IPAQ), short version, previously adapted and validated for use in the Brazilian population [37]. This instrument is composed of eight questions on the duration and frequency of participation in vigorous activities, moderate activities, walking, and sedentary habits in the previous seven days, allowing individuals to be classified into four different categories: very active, active, irregularly active, and sedentary.

### Peripheral muscle strength

Peripheral muscle strength will be evaluated by measuring HGS by dynamometry, using a JAMAR^®^ analog hydraulic dynamometer (Asimow Engineering Co., Los Angeles, CA, USA). To perform the test, the patient must remain in a sitting position, with the dynamometer in the dominant hand, the arm adducted and parallel to the trunk, the shoulder in neutral rotation, the elbow flexed at 90°, and the forearm in neutral position. The evaluator must hold the dynamometer and ask the patient to squeeze it with the greatest possible force, allowing only the wrist and finger joints to move. Three measurements will be made with a maximum interval of 60 s, and the mean of the values found will be considered. To determine the predicted HGS values, an equation specific to the Brazilian population will be used [38].

### Gait speed

GS will be measured by 4mWT. Participants will be instructed to walk at their usual speed, starting from an orthostatic position. Canes and walkers will be allowed if the individual habitually uses such accessories in daily life. GS will be determined by the quotient between the distance (four meters) and the shortest duration of three attempts to walk it (in seconds). The cut-off point will be 0.8 m/s. The test may be performed by any individual capable of walking four meters with the following guidelines [39,40]:

1. The participant must be escorted by the examiner to the designated area, which must be well lit and obstacle-free, and contain clear markings indicating the start and finish lines, zero and four meters, respectively;
2. The participant must be positioned with the feet behind the starting line, just touching it;
3. The participants must be instructed to “walk at their comfortable pace” until a few steps beyond the finish line. The participant must not slow down before the finish line;
4. The examiner must start each attempt with the word “Go” (*“Vá”* in Portuguese);
5. The examiner must start a handheld stopwatch at the patient’s first step after the start line;
6. The examiner must stop the hand-held stopwatch when the patient’s first foot crosses the finish line;
7. The examiner must repeat the procedure three times, allowing sufficient time for recovery between attempts.

### Skeletal muscle mass

Skeletal muscle mass will be evaluated by dual-energy X-ray absorptiometry (DEXA), which consists of measuring the density of the fatty parts of the body and the fat-free mass by means of X-ray emission with two different energy levels (40 and 80 kV).

In this technique, the patient must be positioned at the center of the scanning table, using the table’s center line as a reference for proper alignment, with the head in a neutral position, avoiding any hyperflexion or hyperextension, approximately 3 cm below the upper horizontal line of the scanning area. The patient’s upper limbs must be positioned along the body. If possible, the hands should not touch the legs, and there should be a minimum gap of 1 cm between the upper limbs and torso, without crossing the scan lines. To reduce any movement of the examinee which may affect the accuracy of the examination, Velcro strips will be used to tie the thumbs together with the fingers, knees, and ankles.

For analysis purposes, the main parameter used will be the appendicular skeletal muscle mass (ASMM), both in absolute values and corrected for the square of the height (appendicular skeletal muscle mass index—ASMMi). The following cut-off points will be considered for the diagnosis of reduced skeletal muscle mass: ASMM below 20.0 kg for men or 15.0 kg for women; ASMMi below 7.0 kg/m^2^ and 5.5 kg/m^2^ for men and women, respectively [13].

For volunteers who cannot undergo the composition exam by DEXA, in an attempt to minimize losses in the study, the specific adiposity measurements can be extracted by electric bioimpedance analysis (BIA) using an InBody 720® direct octopolar segmental multifrequency bioimpedance equipment (Biospace, Seoul, South Korea).

### Respiratory muscle strength

Respiratory muscle strength will be measured by manovacuometry, using an analog manovacuometer with a measurement range of −150 to +150 cmH_2_O, (Ventcare, Buri, Brazil) according to the protocols of the American Thoracic Society and the European Respiratory Society [41]. The test will be performed by an examiner who will ask the patient to perform a maximum inspiration from the residual volume followed by a maximum expiration from the total lung capacity for one to two seconds, to quantify the maximum inspiratory pressure (MIP) and maximum expiratory pressure (MEP), respectively. The patient must be in a sitting position and have the nostrils occluded by a nose clip to prevent the closure of the glottis and the pressure generated by the mouth muscles from leading to an overestimated measurement. The highest value of three to five maneuvers will be recorded when successive measurements vary less than 10% [42].

### Cardiopulmonary exercise test

The participants will undergo CPET to assess their aerobic power and capacity, and their hemodynamic, metabolic, and ventilatory responses to exercise. A symptom-limited treadmill (Inbramed, Porto Alegre, Brazil) exercise test will be performed using a ramp protocol, with a duration of approximately 8–12 min. The patients must be encouraged to continue exercise until exhaustion. The test will last a minimum of six minutes followed by a rest phase, with a speed of 1.5 mph and a slope of 2.5% in the first minute.

For measuring the gases, a clip will be placed on the patients’ nose and a mouthpiece with a saliva trap will be connected to a pneumotachograph, which in turn will be connected to a VO2000^®^ gas analyzer (MedGraphics, St. Paul, MN, USA) coupled to a computer. The analysis will be performed using ErgoPC Elite^®^ software (Micromed, Brasília, Brazil). Oxygen uptake (VO_2_), carbon dioxide output (VCO_2_), minute ventilation (VE), and related variables will be calculated at each breath.

The following parameters will be analyzed: peak VO_2_, expressed as a percentage of the predicted value and related to body mass; oxygen uptake at the anaerobic threshold (AT), expressed as a percentage of the predicted value and related to body mass [43]; slope of the ratio of ventilation (VE) to CO_2_ output (VE/VCO_2_ slope); oxygen uptake efficiency slope (OUES), expressed as an absolute value and a percentage of the predicted value [44]; respiratory exchange ratio (RER); maximal systolic blood pressure (SBP_max_); changes in systolic arterial pressure (SBP) from rest to maximal exercise (SBP_Δ_); heart rate reserve (HRR), calculated as the heart rate (HR) change from rest to maximal exercise; chronotropic index (proportion between measured and predicted HRR); HR decrease during the first minute of recovery (HR_rec_); peak exercise oxygen pulse (O_2_P); circulatory power (CP); and ventilatory power (VP).

Peak VO_2_ will be defined as the maximum value detected during the last 20 s of exercise or at the first measurement performed during the rest phase. Participants will be classified according to peak VO_2_ values [45] into the following: A: above 20.0 mL.kg^−1^.min^−1^; B: 16.0–20.0 mL.kg^−1^.min^−1^; C: 10.0–15.9 mL.kg^−1^.min^−1^; and D: below 10.0 mL.kg^−1^.min^−1^. AT will be identified by the ventilatory equivalent method, and 40% of the predicted maximum VO_2_ will be defined as the lower normal limit [46]. The VE/VCO_2_ slope will be calculated during the whole test period.

### N-terminal pro-B-type natriuretic peptide

N-terminal pro-B-type natriuretic peptide (NT-proBNP) levels will be analyzed because this biomarker is secreted in response to increased myocardial filling pressures [1], reflecting the degree of ventricular impairment. Its indication is already well established in the diagnosis and prognostic evaluation of HF [1,47]. NT-proBNP will be measured using immunofluorescence with an automatic analyzer, in which a murine anti-BNP monoclonal antibody captures the NT-proBNP present in the sample. This complex is then bound by a second fluorescent-labeled polyclonal antibody. The NT-proBNP concentration in pg/mL will be obtained by measuring the fluorescence from the standard curve of the device. The dosage is performed within six hours after collection using the Elecsys^®^ system (F. Hoffmann–La Roche, Ltd., Basel, Switzerland). The blood will be collected from the participant’s non-dominant upper limb by a member of the research team.

### Growth differentiation factor-15

Growth differentiation factor-15 (GDF-15) is a pleiotropic cytokine, a member of the beta growth factor family, released by a variable number of cells such as adipocytes and myocytes in response to inflammation, atherosclerosis, ischemia, and oxidative stress [48,49]. Numerous studies have demonstrated that GDF-15 is a good prognostic marker in a wide variety of cardiovascular diseases, including HF [48–50]. In the HF-ACTION Study, GDF-15 added independent prognostic information [49]. GDF-15 measurements will be performed using the Elecsys^®^ system (F. Hoffmann–La Roche, Ltd.).

### Proteomics

Whole plasma samples will be analyzed or they will undergo a depletion process of the 14 most abundant proteins of the plasma using the Human 14 Multiple Affinity Removal Column (4.6 × 100 mm; Agilent Technologies, Inc., Santa Clara, CA, USA), according to the manufacturer’s indications. The samples will then be fractionated on adsorbent columns (Sep-pak C-18, 20 mg; Waters Corporation, Milford, MA, USA). The protein concentration will be determined by a fluorometric assay using the Qubit^®^ reagent (Invitrogen^®^ Qubit^®^ Quantitation Kit; Thermo Fisher Scientific, Inc., Waltham, MA, USA), according to the manufacturer’s instructions. Proteins will be reduced with 10 mM dithiothreitol at 30°C for 60 min and alkylated with 40 mM iodoacetamide for 30 min at room temperature in a place protected from light. Before the trypsinization step, the samples will have their pH adjusted to 8.0 with 0.1% sodium hydroxide and will be incubated with trypsin (Promega Corporation, Madison, WI, USA) at a 1:50 ratio (μg of trypsin:μg of protein).

The digestion step will be carried out for 18 h at 37°C. The reaction will be stopped by the addition of 10% trifluoroacetic acid to obtain a final concentration of 1%. When necessary, desalting of the samples will be performed using stage-tip columns produced with Poros™ R2 reverse phase resin (Thermo Fisher Scientific, Inc.). Finally, samples will be vacuum centrifuged for drying and resuspended in 0.1% formic acid for nanoscale liquid chromatographic tandem mass spectrometry (nLC-MS/MS) analysis. Label-free quantitative analysis will be performed using the chromatographic regions of the identified peptides to infer protein abundance. In addition, a quantification methodology that correlates peptide abundance with the number of peptide spectral matches obtained in their identification will also be applied.

Fractionation of the samples will be performed offline on a UFLC system (Shimadzu Corporation, Kyoto, Japan) using a Gemini C18 reversed phase column (250 × 4.6 mm, 5 μm; Phenomenex, Inc., Torrance, CA, USA). Fractionation will occur at basic pH by applying an increasing gradient of 90% acetonitrile and 20 mM ammonium bicarbonate. Gradient, flow, and run time will be optimized according to sample complexity.

The processed samples will be suspended in 0.1% formic acid and quantified by the method described using the Qubit^®^ reagent (Qubit^®^ Quantitation Kit, Thermo Fisher Scientific, Inc.). Next, samples will be diluted to a final concentration of 0.5 μg/μl and 2 μg of peptides will be analyzed in an EASY-nLC 1000 system (Thermo Fisher Scientific) coupled online to a Q Exactive Plus mass spectrometer (Thermo Fisher Scientific). The peptides will be injected into a 3-cm (length) and 100-μm (inner diameter) pre-column packed with 5-μm ReproSil-Pur C18 resin (Dr. Maisch HPLC GmbH, Ammerbuch, Germany), then fractionated on a 25-cm (length) and 75-μm (inner diameter) PicoFrit column (New Objective, Inc., Littleton, MA) packed with 3-μm ReproSil-Pur C18 resin (Dr. Maisch HPLC GmbH).

Elution will be performed by applying a gradient with 95% acetonitrile and 0.1% formic acid solution, which will be adjusted according to the complexity of the samples. The data-dependent analysis (DDA) method will be used, in which the instrument will be configured to select the 20 most intense peptides for the fragmentation step in a high-energy collision dissociation (HCD)-type collision cell using a Normalized Collision Energy™ (NCE; Thermo Fisher Scientific) of 30.

For the full scan analysis (MS1), the Orbitrap mass analyzer resolution will be 70,000 (m/z 200) with automatic gain control (AGC) 10^5^ and a maximum injection time of 50 ms. To obtain fragmentation spectra (MS2), the Orbitrap resolution will be 17,500 (m/z 200) with AGC 10^5^, a maximum injection time of 100 ms, and an isolation window of 2 m/z. The dynamic exclusion time will be set to 45 s.

After the characterization of the total proteome and evaluation of the results, the altered proteins (candidate biomarkers) will be validated through an analysis of the samples by target-directed proteomics strategies. The selected reaction monitoring (SRM) method will be used, and the analyses will be performed in a triple-quadrupole mass spectrometer. Skyline software version 4.2 (MacCoss Lab, University of Washington, Seattle, WA, USA) will be used for the definition of the unique peptides of each protein of interest. Isotope-labeled peptides will be synthesized for use in the optimization of the parameters of the analysis method in confirming the identification of the targets, and in their relative quantification. The digested plasma samples will be analyzed in an EASY-nLC 1000 system (Thermo Fisher Scientific) coupled online to a TSQ Quantiva mass spectrometer (Thermo Fisher Scientific). The pre-column, column, and gradient parameters will be the same as described previously. The instrument will be configured according to the parameters defined in the method development.

The data obtained in the DDA will be analyzed with Proteome Discoverer software, version 2.1 (Thermo Fisher Scientific). The PSM method will be applied to identify the peptides using the Sequest HT algorithm. The search will be performed in the *Homo sapiens* database or in that of the organism of interest deposited in Uniprot https://www.uniprot.org/), considering the carbamide methylation of cysteines as a fixed modification and the oxidation of methionines as a dynamic modification.

The results obtained will be analyzed using biocomputing tools such as DAVID (available at: https://david.ncifcrf.gov/) and the Kegg Pathway Database (available at: https://www.genome.jp/kegg/pathway.html). Quantitative analysis will be performed using Perseus software (Max Planck Institute of Biochemistry, Munich, Germany). The data analysis of biomarker candidates by the SRM method will be performed with Skyline software, version 4.2 (MacCoss Lab). For the quantification of targets, the chromatographic regions of the identified peptides will be normalized by the area of their respective isotope-labeled peptides.

### Genomics

Genomic DNA will be obtained from saliva samples collected with Oracollect•DNA OCR-100 swabs (DNA Genotek, Kanata, ON, Canada), according to the manufacturer’s instructions. Briefly, 500 μl of the sample will be mixed with prepIT•L2P purification reagent (DNA Genotek) for cell lysis and precipitation of protein content. The supernatant will be collected and subjected to two steps of alcohol precipitation for DNA separation. At the end of the process, the DNA will be eluted in nuclease-free water and quantified by fluorescence spectroscopy using the Qubit^®^ dsDNA HS assay kit (Thermo Fisher Scientific) in a Qubit^®^ fluorometer. The quality of the DNA will be evaluated using a NanoDrop™ One spectrophotometer (Thermo Fisher Scientific).

Library preparation for whole-exome sequencing will be performed based on the hybridization and capture enrichment method using Illumina DNA Prep with Enrichment and Illumina DNA Prep reagents (Illumina, Inc., San Diego, CA, USA). Briefly, genomic DNA from each sample will be fragmented and barcodes (IDT for Illumina UD Indexes Set A) will be added at their extremities. After a purification step, the pre-enriched libraries will be quantified in the Qubit^®^ fluorometer (Thermo Fisher Scientific) and the samples will be pooled. Biotin-linked, human exome-specific probes (Illumina Exome Panel) will be used to hybridize the DNA, which will be captured using streptavidin-linked magnetic beads. Finally, the enriched library will be amplified and purified, and quantity and quality parameters will be determined in the Qubit^®^ fluorometer and in a Bioanalyzer automated electrophoresis device (Agilent). All library preparation will be automated in an EpMotion 5075 automated liquid preparation system (Eppendorf SE, Hamburg, Germany) customized for the Illumina DNA Prep with Enrichment protocol (Illumina). Libraries will be sequenced on a NovaSeq 6000 sequencing platform (Illumina) using S4 cartridges.

The analysis of the FASTQ file will be performed on the BaseSpace platform (Illumina) using the DRAGEN Enrichment application (Illumina). The VCF file generated will be loaded into the Variant Interpreter (Illumina) for filtering steps according to the following criteria: list of genes with robust evidence of relation with the respective disease, following the Clinical Genome Resource (ClinGen) guidelines; population frequency of the variant in the gnomAD database; variant type; consequences of the variant; and quality metrics. Other data filtering steps can be done in the R statistical platform (The R Foundation, Vienna, Austria). The variants found will be classified according to the guidelines of the American College of Medical Genetics (ACMG) and ClinGen.

### Machine learning

Machine learning models will be created to identify molecular mechanisms that make an individual more prone to sarcopenia and how to identify such mechanisms from genomic and proteomic data. To this end, four types of machine learning methods will be investigated. The first is (i.) “classical” machine learning, which operates on a set of values and their attributes. Attribute selection and identification algorithms will be considered to identify which attributes prove to be most relevant for a given diagnosis [51]. In parallel, models based on (ii.) deep learning from unstructured data will be trained. In this case, methods that induce models from abdominal computed tomography (CT) images will be used, including classification and segmentation models [52,53]. As the data obtained from the sample include several modalities, namely structured data in attribute-value format and unstructured data, models will also be created from (iii.) multimodal learning [54]. In this case, both clinical and laboratory examinations, as well as CT images and textual reports, will be used together to train a model. It is expected that the different modalities can collaborate for the generation of more accurate predictive models from early data fusion, in which attributes are arranged in the same representation space, as well as taking advantage of late fusion, in which models are trained from each modality and their outputs are combined. Finally, since exome data have many relationships among their components, models (iv.) based on network analysis and relational learning [55–57] will be trained to induce and identify patterns from the co-expression of genes and proteins for an analysis of the causative and influencing factors of sarcopenia.

In all cases, model training will be performed from historical data using a cross-validation procedure with k partitions. Thus, at each training iteration, k−1 partitions are used for model training and generation, while the remaining partition is used to test the predictive abilities and the generalization of the model. This ensures that all examples are used for training and testing at least once, but in different iterations, and reduces the chance of the test set not being representative due to random choices.

For model hyperparameter adjustments—that is, parameters that guide model generation, a validation set internal to the training will be used. The trained model will be evaluated on the test set using standard metrics, which include calculation of accuracy, precision, revocation, and F-measure for discrete outputs, and mean squared error for continuous outputs. Furthermore, graphs and corresponding values of the areas under the receiver operating characteristics (ROC) curve will be used to understand the behavior of the values predicted by the models in different classes. Furthermore, the analysis of the models generated will include the use of methods of explanation and interpretation to mitigate the black-box aspects of some models and aid in the understanding and collaboration of experts.

### Statistical considerations

The data will be submitted to a normality test (Shapiro–Wilk test) and a homogeneity test (Levene test). Continuous quantitative variables will be expressed as means ± standard deviations or medians and interquartile ranges, and categorical variables as absolute values and percentages. Comparisons between groups will be performed using either the unpaired two-tailed Student t-test (normal distribution) or Mann–Whitney test (non-normal distribution). Fisher’s exact test will be used in the comparison of categorical variables. Pearson’s correlation will be used for the association between the variables studied.

Interval variables will be described by the mean, standard deviation, and five-number summary (minimum and maximum values and the 25th, 50th, and 75th percentiles). Nominal variables will be described by their counts and proportions. Ordinal variables will be described either by their proportions or by the five-number summary, depending on the number of levels.

The present study has an adaptive approach and will have two parts: validation of the control group and analysis of the case group. Considering a 20% prevalence of sarcopenia in a population with HF and a clinically significant relative odds ratio (OR) difference of 35%, 150 patients per study arm will be needed to ensure a power of 80% with 95% confidence. For the validation of the control group, a total of 150 patients will be selected from a population of 300 patients with HFrEF, of which 30 will be with sarcopenia according to the established criteria. After that, the LVEF will be evaluated by Simpson’s method and the OR between sarcopenia and reduced LVEF will be calculated. If the OR is close to 2.3, there will be no need to correct the sample size and the study will proceed to the construction of the case group. For the case group, 45 patients with an alteration in the exome that causes a relative alteration of 35% in the OR of the control group will be necessary. However, the prevalence of this possible alteration is unknown. Estimating that this alteration is in 30% of the patients with HFrEF and sarcopenia, then 150 patients with HFrEF will be necessary to find these 45 with the alteration.

Effect inference will be made using a logistic regression model with the dependent variable of reduced LVEF and the presence of regressors including sarcopenia, exome alteration, and an interaction term.

The association between the presence of sarcopenia and/or dynapenia with the risk of clinical events and death, quality of life, cardiorespiratory capacity, ventilatory efficiency, and respiratory muscle strength will be determined by means of logistic regression models (for analyses with cross-sectional data), Cox regression models (for longitudinal analyses of clinical outcomes), or generalized linear models (for longitudinal analyses of continuous variables). The significance level established will be p < 0.05.

### Safety considerations

There is a very rare risk of complications during CPET. For the participant’s safety, this test will be performed by a physician trained in advanced cardiac life support (ACLS). This professional will follow all the steps of the exam and will be responsible for any necessary care. In addition, the ergometry services of the participating institutions have the necessary equipment to treat and solve these emergencies according to the ACLS protocols, which will be adopted if necessary. It must be noted that CPET is considered an essential test in the diagnostic evaluation, risk stratification, and evolutionary follow-up of individuals with HF, allowing a more appropriate and accurate determination of functional capacity, which is why it is the guiding method in the decision on eligibility for heart transplantation. Thus, CPET will be very useful for the evaluated participants, regardless of the objectives of the study.

The risk for the HGS test is only self-limited local pain after the measurement. There is a small risk of falling in the GS test, but the participant will always be closely accompanied by one of the researchers and can use a cane or walker if they use them regularly. For the respiratory strength test, there is a possibility of self-limited fatigue after the test. DEXA is a painless, simple, fast, non-invasive exam whose only risk is the same as that of a conventional X-ray, namely exposure to radiation, which, however, will be extremely small and brief. Contraindications for DEXA are pregnancy, body mass above 120 kg, and contrasted exams (iodine or barium) of the digestive or urinary tract in the previous two weeks, because the contrast may interfere in the results. The risks for blood collection are only hematoma, bleeding, and pain at the site of venipuncture. To avoid hematomas, the blood samples will only be collected by healthcare professionals with experience in venous puncture for this purpose. After venous puncture, soft-to-moderate compression will be applied on the puncture site for 5–10 min until no hemorrhage is observed.

If any complication happens to the participants during the study, they will receive all the treatment that is necessary and available.

By using medical records to obtain clinical data, the research participants may be exposed to a small risk of breach of confidentiality of their identity. The researchers, however, will be the only ones to have access to the password-protected data and will take all necessary measures to preserve the privacy of the participants.

### Data storage and management

The research data will be collected, managed, and disseminated through the Research Electronic Data Capture (REDCap) platform (Vanderbilt University, Nashville, TN, USA), and password-protected access will only be granted to participating researchers.

### Status and timeline of the study

Protocol version February 2023. Recruitment began on September 2022. Trial recruitment is estimated to be completed in October 2024.

## Discussion

Individuals with chronic HF can be divided into two broad categories based on LVEF. Although this classification may be considered somewhat arbitrary, patients in these two groups have many significant differences [58]. HFrEF is characterized by more profound abnormalities in systolic function than those seen in HF with preserved LVEF, usually with progressive chamber dilatation and eccentric remodeling of the left ventricle. Currently, HFrEF is most commonly defined by LVEF equal to or less than 40% [59].

The identification of prognostic factors should be part of the initial evaluation of any individual with HF. The factors most often used to predict survival in patients with HFrEF include the New York Heart Association (NYHA) functional class; the magnitude of LVEF reduction; concomitant diastolic dysfunction, as established by the mitral flow velocity pattern on Doppler echocardiography; right ventricular dysfunction; reduced peak VO_2_ value on CPET; markers of reduced tissue perfusion, such as low mean arterial pressure, decreased arterial pulse pressure, renal failure (creatinine clearance below 60 mL/min and a diminished response to diuretics); and elevated plasma levels of natriuretic hormones such as B-type natriuretic peptide (BNP) and NT-proBNP [60].

The prevalence of primary sarcopenia varies depending on the population assessed and the criterion adopted. It may affect up to 29% of community-dwelling elderly and 14%–33% of institutionalized elderly [61]. A loss of lean body mass of 1%–2% per year in the lower limbs and a loss of muscle strength between 1.5% and 5% per year are estimated from the age of 50 onwards [62–65].

Dyspnea, muscle fatigue, and intolerance to effort are the most characteristic clinical manifestations of HF [1]. These symptoms, however, cannot be totally attributed to central cardiovascular alterations. There are peripheral mechanisms that contribute to the symptoms’ appearance and persistence, and make up HF-related skeletal myopathy [66–69] including, among other factors, changes in the percentage of type I (oxidative, slow-twitch) and type IIb (glycolytic, fast-twitch) muscle fibers; reduced capillary density; decreased muscle metabolism, with rapid depletion of high-energy phosphates and rapid decline in muscle pH on exercise; lower mitochondrial density and mitochondrial oxidative enzyme activity; and muscle atrophy [66].

Multiple mechanisms may explain the evolution of patients with HF to sarcopenia, but some of them stand out due to the low-output state and the consequent neurohumoral regulation: reduced muscle blood flow, endothelial dysfunction, malnutrition, physical inactivity, inflammation, oxidative stress, apoptosis, autophagy, activation of the ubiquitin-proteasome system, and hormonal changes [70,71]. As a result, an imbalance between muscle anabolism and catabolism can be observed, as well as mitochondrial dysfunction, muscle fiber transformation, and connective tissue infiltration into the muscle [70]. Finally, it is worth considering an interesting conjecture by von Haehling that there could be a gradual evolution from sarcopenia, in which only the skeletal muscle mass is reduced, to subsequent cachexia, in which associated reductions of bone mass and adipose tissue occur, leading to weight loss [72].

For these reasons, it is clear that dynapenia and sarcopenia can aggravate the condition of patients with HFrEF. The questions this study intends to investigate are whether there are molecular mechanisms that make the individual with HFrEF more prone to dynapenia and/or sarcopenia, and if such mechanisms can be identified through molecular markers (by genomic and proteomic analysis) that can predict which individuals with HFrEF will present concomitant dynapenia and/or sarcopenia.

Genomic and proteomic analysis in individuals with HFrEF with and without dynapenia and/or sarcopenia may indicate an impairment of different signaling pathways. This allows the definition of distinct pharmacological or non-pharmacological therapeutic strategies to mitigate or reverse the impairment of these pathways, such as nutritional intervention and individualized physical exercise programs, with the ultimate goal of obtaining clinical and functional improvement and, consequently, positive effects on the patients’ quality of life and prognosis.

Emami *et al*. found a sarcopenia prevalence of 21.3% in men with HF using ASMM evaluated by DEXA as the sole diagnostic criterion with a cut-off point of 7.26 kg/m^2^ [28]. The present study will make use of more recent criteria for the diagnosis of sarcopenia that mainly emphasize muscle strength, and, at least initially, a similar prevalence is estimated, that is, around 20%.
